# Evaluation of the Antioxidant Status of Ethanolic Extract of *Hypericum perforatum* and Its Effect on Performance, Immune System, Antioxidant Status and Plasma Biochemical Indices of Broiler Chickens

**DOI:** 10.1002/vms3.71050

**Published:** 2026-06-28

**Authors:** Hojjat Seraji‐Kopkan, Seyyed Javad Hosseini‐Vashan, Nazar Afzali, Mohammad Hassan Namaei

**Affiliations:** ^1^ Animal Science Department University of Birjand Birjand Iran; ^2^ Department of Laboratory Science, Faculty of Paramedical and Rehabilitation Sciences Mashhad University of Medical Sciences Mashhad Iran

**Keywords:** abdominal fat, blood biochemical parameters, *Hypericum perforatum*, immune system, malondialdehyde

## Abstract

**Background:**

Plant extracts contain bioactive compounds that have positive effects on the immune response and antioxidant system in living cells. St. John's wort (*Hypericum perforatum*) is a medicinal plant that contains hypericin, hyperforin and pinene, which have antiviral, anti‐inflammatory, antimicrobial and immune stimulating effects.

**Objectives:**

This experiment was conducted to evaluate the antioxidant activity of *Hypericum perforatum* ethanolic extract (HPEE) and its effect on growth performance, abdominal fat, immune response and blood biochemical indices of broiler chicks.

**Methods:**

A number of 200‐day‐old male Ross‐308 broiler chickens were randomly assigned to four treatments with five replications and 10 birds each. Dietary treatments included 0, 150, 300 and 450 mg/kg HPEE. Body weight, feed intake and feed conversion ratio were recorded in broiler chickens during the starter (0–10 days of age), grower (11–24 days of age) and finisher (25–42 days of age) periods. To evaluate antioxidant activity, immune system, blood biochemical traits and relative weight of visceral and lymphatic organs, two birds from each replicate were sacrificed and blood was gathered at 24 and 42 days of age.

**Results:**

Dietary treatments did not affect growth performance including feed intake, body weight and feed conversion ratio. On Day 24, the relative weight of abdominal fat in birds fed HPEE was reduced compared to the control (*p* < 0.05). Relative crop weight increased in birds fed the 450 mg/kg HPEE, compared to the control diet on Day 24 (*p* < 0.05). The lowest concentration of malondialdehyde (MDA) was observed in 450 mg/kg HPEE, which was significantly lower than control (*p* < 0.05). In addition, HPEE increased the total antibody titer and IgM titer against sheep red blood cells (SRBC), compared to control (*p* < 0.05). Blood indices including cholesterol, LDL and glucose were reduced by HPEE diets at 42 days. HPEE caused a decrease in plasma aspartate aminotransferase (AST) enzyme activity.

**Conclusion:**

HPEE supplementation in broiler diets can improve the immune system and may reduce abdominal fat, serum AST, alanine aminotransferase (ALT) enzyme activities and MDA concentration in broiler chickens, without having an adverse effect on growth performance and carcass characteristics of broiler chickens.

## Graphical abstract

*Hypericum perforatum* ethanolic extract (HPEE) at the levels of 0, 150, 300 and 450 mg/kg were used and dietary treatments did not affect growth performance including feed intake, body weight and feed conversion ratio. The HPPE reduced the abdominal fat and improved meat quality in broilers. HPEE increased the total antibody titer and IgM titer against sheep red blood cells (SRBC). Blood indices including cholesterol, LDL, glucose, malondialdehyde (MDA) concentration and plasma AST enzyme activity were reduced by HPEE diets.

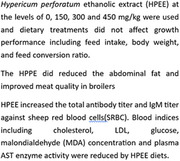

## 1 Introduction

*Hypericum perforatum* L. (*HPE*) belongs to the Hypericaceae family and has more than 469 species that exist around the world (Cirak et al. 2010). This plant originates from Europe, West Asia and North Africa, and is known as one of the oldest herbal medicines used in medical sciences (Axarlis et al. 1998; Flausino et al. 2002; Mattace et al. 2002; Brenner et al. 2000; Rabanal et al. 2005). There are several important bioactive compounds in *HPE*, the main active components of which are hyperforin (phloroglucinol derivative), hypericin (anthrone nephroid) and quercetin (flavonoid) (Barnes et al. 2001). In addition to healing burns, bruises, swellings and wounds, *HPE* and other species have antiviral, antidepressant, antioxidant (Jakovljević et al. 2000) and antimicrobial properties, and protect the liver from collateral damage (Spiteller et al. 2008).

Antioxidants are biologically active compounds that protect the body against diseases and damage caused by reactive oxygen species (ROS), reactive nitrogen and reactive chlorine species (Zaveri 2006; Shahidi and Naczk 2004; Pekkarinen et al. 1999). In the body, free radicals and these reactive species can damage the lungs, heart, cardiovascular system, kidneys, liver, eyes, skin and muscles, and can accelerate the aging process of brain cells (Pekkarinen et al. 1999). The antioxidant activity of extracts containing high concentrations of flavonoids (FCHP) from the plant *Hypericum* was observed in China. FCHP is a potent inhibitor of DPPH and superoxide radicals (Zhou et al. 2004). Therefore, it was suggested that *Hypericum* extract be added to chicken water to reduce losses and feed conversion ratio (FCR) (Feizi and Nazeri 2011).

*HPE* extract is known to be hypocholesterolemic because intraperitoneal injection in mice reduced blood cholesterol, triglycerides and total protein (Landy et al., 2012). *Hypericum* extracts also reduced the haemoglobin and red blood cell diameter and increased the percentage of white blood cells and neutrophils. Some researchers have reported that *Hypericum* extract improves the immune system of mice (Aghili et al. 2014). Behroozilak et al. (2025) reported that *HPE* reduces blood cholesterol, LDL, malondialdehyde (MDA) concentration and relative abdominal fat weight in broiler chickens without any influence on growth performance. However, they reported that *HPE* supplementation to broiler diets increased the total antioxidant capacity and antibody titer and relative thymus weight in broiler (Behroozilak et al. 2025). It was also reported that *HPE* improved body weight gain, antibody titer against Newcastle disease and *Lactobacillus* counts in microflora of broiler chickens (Ihan et al. 2024). The body weight, FCR, antioxidant status and immune response in birds fed water extract of *HPE* improved (Seraji‐Kopkan et al. 2015). Despite these findings, knowledge is still limited regarding the effects of *HPE* on growth performance traits in poultry production, especially broilers. Therefore, the aim of this study was to evaluate the antioxidant activity of ethanolic extract of *HPE* and its effect on growth performance, carcass characteristics, immune responses, serum biochemical indices and liver enzymes activities in broiler chickens.

## 2 Materials and Methods

### 2.1 Birds, Diets and Husbandry

A total of 200 1‐day‐old male Ross 308 broiler chicks were weighed and randomly assigned to four treatments with five replications and 10 chicks each in a completely randomized design. Then, the chicks are reared for 6 weeks in a pen with the dimensions of 80 × 120 × 120 cm. Dietary treatments included 0, 150, 300 and 450 mg/kg *Hypericum perforatum* ethanolic extracts (HPEE). Feed and water were supplied ad libitum. Diets were used based on the age recommended by the Ross 308 catalogue for the starter (0–10 days of age), grower (11–24 days of age) and finisher (25–42 days of age) periods (Aviagen n.d.) (Table 1). The photoperiod schedule consisted of 23:1 h light:dark.

**TABLE 1:** The ingredient and chemical composition of basal starter, grower and finisher diets.

Ingredients (g/kg)	Starter	Grower	Finisher
Corn	551.2	574.2	627.0
Soybean meal	360.0	348.3	292.0
Soybean oil	15.3	35.7	40.1
Fish meal	40.0	—	—
CaCO_3_	12.3	14.8	14.1
DCP	10.3	15.8	15.3
NaCl	4.00	4.00	4.00
DL‐methionine	1.9	2.1	1.8
Mineral premix^a^	2.5	2.5	2.5
Vitamin premix^b^	2.5	2.5	2.5
**Calculated composition**			
Metabolisable energy (kcal/kg)	3100	3120	3200
Crude protein (g/kg)	230.0	205.0	185.0
Calcium (g/kg)	10.5	10.0	9.5
Av. phosphorus (g/kg)	5.2	5.0	4.7
Sodium (g/kg)	1.9	1.7	1.7
Arginine	15.7	14.0	12.5
Lysine (g/kg)	13.6	11.4	9.9
Meth. + cysteine (g/kg)	9.2	8.2	7.4
Threonine	9.2	8.1	7.4
Tryptophan	2.8	2.6	2.3

^a^Note that 2.5 kg of vitamin premix contained 2700 mg retinal, 400 mg calcidiol, 18 g tocopheryl acetate, 2000 mg menadione, 1800 mg thiamine, 6600 mg riboflavin, 10 g niacin, 30 g calcium pantothenate, 3 g pyridoxine, 1 g folic acid, 15 mg cobalamin, 250 g choline chloride and 100 mg biotin.

^b^Note that 2.5 kg of trace mineral premix contained 100 g Mn, 75 g Fe, 9 g Zn, 20 g Cu, 1.1 g I and 180 mg Se.

### 2.2 Extract Preparation

St. John's wort (*HPE*) was collected during the flowering season from the Hezar Masjid Mountains located in Dargaz, Khorasan, Iran. Aerial parts were harvested between 10:00 and 14:00 h in clear and sunny days. Then, the samples were air‐dried under standard conditions (30°C–35°C) and powdered. Note that, 10 g of the fine powder was separately added to 100 mL of ethanol 96% and gently mixed for 72 h to obtain a good quality extract. Then, the mixture of solvent and plant was filtered through filter paper (Whatman No. 1) and the crude extract was obtained. Finally, the solvent was slowly evaporated in vacuum distillation apparatus (rotary evaporator) at 80°C for 1 h to produce a concentrated extract (Jafari et al. 2021; Forbes et al. 1990).

The chemical composition of HPEE was analysed by Varian CP‐3800 GC/MS and DB‐5 capillary column (30 m × 0.25 mm, with a coating thickness of 0.25 µm) and a Varian Saturn 2000 ion trap mass detector. The injector and transfer line temperatures were 220°C and 240°C, respectively. Also, the oven temperature was set at a programmed temperature of 60°C–240°C at a temperature rate of 3°C per minute. The carrier gas was helium at a rate of 1 mL/min. Triple injections of 0.2 mL sample were used with a split ratio of 1:30. Then, the component was identified based on retention times with authentic samples; their linear retention towards C5‐C24 *n*‐alkanes and two databases were compared: NIST 98 (National Institute of Standards and Technology) library and the Chemistry Data System (Wiley VCH Verlag GmbH and Co. KGaA, Weinheim, Germany). In addition, GC peak areas were used to calculate the percentage of components (Mclafferty 2009).

### 2.3 Growth Performance

Broilers were weighed on Days 1, 10, 24 and 42. Feed intake and weight gain were recorded, and FCR was calculated at the starter (1–10 days), grower (11–24 days) and finisher (25–42 days) period of the experiment. In addition, mortality was recorded daily. Weight gain, feed intake and FCR was computed and corrected based on hen day.

### 2.4 Carcass Characteristics

On Days 24 and 42, after 4 h of starvation, two chicks from each experimental unit (a total of eight birds from each treatment) with a weight close to the group average were randomly selected and slaughtered (sacrificed). Carcass yield was calculated by dividing the net carcass weight (carcass without skin) by the live weight of the bird. Abdominal fat, gizzard, crop, liver and pancreas were removed, weighed and calculated as a percentage of live body weight. The relative weight of lymphatic organs (spleen and bursa of Fabricius) was also measured.

### 2.5 Immune System

Note that 0.4 mL of sheep red blood cells (SRBC) was injected intravenially into the wing vein of eight chickens (two chickens per replicate) in each treatment on Days 24 and 37. Five days after each injection, blood was collected from the chickens and antibody titers and immunoglobulin production in response to SRBC were assessed by the haemagglutination method (Nelson et al. 1995).

### 2.6 Blood Biochemical Indices

After 12 h of fasting, two broiler chickens were selected from each replicate and blood samples were taken via wing vein and collected without heparin tubing on Days 24 and 42. Blood samples were centrifuged for 15 min at 2000 rpm to separate sera (Hettich Universal Centrifuge, Germany). Serum samples were also analysed separately using laboratory test kits manufactured by Pars Azmoun Company and using a GesanChem autoanalyser (Gesan, Italy) to determine total cholesterol, high‐density lipoprotein (HDL), low‐density lipoprotein (LDL), triglycerides and glucose.

### 2.7 Antioxidant Status

MDA concentration was determined as a thiobarbituric acid reactions score (TBARS) and is used to assess lipid peroxidation. At low pH and high temperature (100°C), MDA binds to TBA to form a red complex that can be measured at a wavelength of 532 nm. Increased red pigment formation is associated with oxidative rancidity of fat. To measure MDA concentration, 2 mL of trichloroacetic acid and 2 mL of an aqueous TBA solution was added to 1 mL of plasma samples and shacked in hot water in Benmari equipment for 10 min. After cooling and adding of butanol, the mixture was centrifuged. The superficial layer of the mixture was taken and read at 532 nm with a spectrophotometer (Ottolenghi 1959; Yoshioka et al. 1979).

### 2.8 Statistical Analysis

The data were organized in Excel, then sorted and classified. All percentage and non‐numerical data were subjected to arcsine transformation prior to statistical analysis. Data were analysed using analysis of variance (ANOVA) based on a completely randomized design via the general linear model (GLM) procedure in SAS software (SAS 2018). Tukey's test was used to compare group means at a significant level of *p* < 0.05.

## 3 Results

### 3.1 The Chemical Composition of HPEE

Thirty‐four volatile elements were identified from the main components in HPEE essential oil by GC/MS analysis. The main components in HPEE are α‐pinene (11.86 %), β‐pinene (5.56%), α‐muurolene (6.56%), 1‐dodecanol (5.95%), undecane (5.31%), α‐cedrene (4.8%), β‐ocimene (4.56%), myrtenol (4.24%) and tridecane (4.19%). In addition, myrcene, limonene, 2‐methyl decane, thymol, geranyl acetate, erlangen, copaene, caryophyllene, cadinene, aromadendrene, germacrene, cadinene, calacorne, spathulenol, 2,6nonadienal, carotol, humulene‐epoxide II, eplerenone, phytol, sesquiterpene and α‐bisabolol are other components identified in HPEE in small amounts by GC/MS (Table 2).

**TABLE 2:** Percentage of chemical composition of *Hypericum perforatum* L. ethanolic extract.

No	Components	RI (retention indices)	Area (%)
1	ND	801	—
2	α‐Pinene	939	11.86
3	Nonane, 3‐methyle	966	2.03
4	β‐Pinene	979	5.56
5	β‐Myrcene	991	1.92
6	α‐Phelendrene	1006	0.34
7	Limonene	1025	0.53
8	β‐Ocimene	1042	4.56
9	Decane, 2‐methyle	1060	3.56
10	Undecane	1100	5.31
11	Myrtenol	1200	4.24
12	Thymol	1282	2.46
13	Tridecane	1291	4.19
14	Geranyl acetate	1364	1.99
15	α‐Ylangene	1371	1.30
16	α‐Copaene	1377	2.01
17	α‐Cedrene	1401	4.80
18	β‐Caryophyllene	1411	2.01
19	Aromadendrene	1429	2.41
20	1‐Dodecanol	1463	5.95
21	Germacrene	1472	6.56
22	α‐Muurolene	1501	3.45
23	ᵧ‐Cadinene	1524	1.22
24	α‐Cadinene	1542	0.87
25	α‐Calacorene	1547	0.76
26	Spathulenol	1565	2.72
27	(E,Z)‐2,6‐nonadienal	1599	2.34
28	Carotol	1603	0.21
29	Humulene‐epoxide II	1608	1.46
30	β‐Oplopenone	1613	0.63
31	Phytol	1647	3.53
32	Sequiterpene	1668	2.09
33	α‐Bisabolol	1686	0.60
34	Germacra‐4(15),5,10(14)‐triene‐1‐α‐ol	1689	1.25

*Note*: Total identified (%).

Abbreviation: ND, not detected.

### 3.2 Performance and Carcass Traits

Table 3 shows data on the effect of HPEE on growth performance traits involving average body weight, feed intake and FCR in broiler chickens. The mean body weight of broilers at the beginning of the experiment (1 day old) was close together and there was no significant difference among the experimental groups. Then, the effect of HPEE on weight gain and FCR were not statistically different. Also feed intake throughout periods of grower and finisher periods did not differ significantly among treatments. However, during starter period, birds fed 150 mg/kg HPEE had higher feed intake than the control group (*p* < 0.05).

**TABLE 3:** Effect of experimental diets of ethanolic extract of *Hypericum perforatum* L. (HPEE) on performance indices of broiler chickens.^a^

Concentration of HPEE (ppm)					
Variable	0	150	300	450	*p*‐value^b^
**FI (gram)**					
0–10	202.97 ± 3.17b	215.50 ± 1.98a	212.37 ± 3.90ab	213.40 ± 6.30ab	0.011
11–24	1037.48 ± 16.75	1046.53 ± 12.91	1025.25 ± 17.19	1021.33 ± 26.20	0.774
25–42	2847.50 ± 34.20	2947.20 ± 163.12	2838.10 ± 121.98	2697.10 ± 32.22	0.364
0–42	4057.40 ± 46.56	4186.20 ± 159.53	4026.30 ± 138.57	3890.80 ± 59.08	0.303
**BW (gram)**					
0–10	160.25 ± 2.95	175.25 ± 7.46	163.25 ± 6.00	169.82 ± 6.18	0.546
11–24	718.00 ± 35.69	708.89 ± 28.07	659.00 ± 29.35	668.50 ± 26.95	0.237
25–42	1378.1 ± 39.29	1414.8 ± 43.21	1385.35 ± 38.46	1361.58 ± 36.27	0.648
1–42	2256.90 ± 41.93	2328.80 ± 110.17	2207.60 ± 32.24	2199.90 ± 72.27	0.224
**FCR**					
0–10	1.27 ± 0.04	1.23 ± 0.01	1.29 ± 0.04	1.26 ± 0.01	0.573
11–24	1.45 ± 0.04	1.48 ± 0.08	1.55 ± 0.06	1.53 ± 0.06	0.637
25–42	2.07 ± 0.05	2.08 ± 0.06	2.05 ± 0.05	1.99 ± 0.05	0.546
0–42	1.79 ± 0.02	1.80 ± 0.03	1.82 ± 0.04	1.76 ± 0.04	0.842

*Note*: Values in rows with different letters differ significantly (*p* ≤ 0.05).

Abbreviations: BW, body weight; FCR, feed conversation ratio (g/g); FI, feed intake (g per bird).

^a^Results are mean ± SEM with *n* = 5 per treatment.

^b^All variables were analysed using Tukey's range test.

Table 4 shows the effect of HPEE on relative carcass weight and digestive tract segment at 24 and 42 days of age. Broilers fed 150 mg/kg HPEE had lower abdominal fat percentage than the control group on Day 24 (*p* < 0.05). Therefore, HPEE did not change the relative weight of carcass, liver, pancreas, proventriculus, gizzard and crop except for the relative crop weight at 24 days when chickens fed 450 mg/kg HPEE had significantly higher crop percentage than the control.

**TABLE 4:** Effect of experimental diets of ethanolic extract of *Hypericum perforatum* L. (HPEE) on carcass yield and internal relative organ weight of broilers at 24 and 42 days.^a^

Concentration of HPEE (ppm)					
Relative organ weight (%)	0	150	300	450	*p*‐value^b^
**24 days**					
Carcass (%)	56.58 ± 1.16	54.17 ± 1.16	53.33 ± 1.22	53.47 ± 1.09	0.22
Abdominal fat (%)	2.01 ± 0.23a	1.12 ± 0.14b	1.42 ± 0.11ab	1.61± 0.20ab	0.02
Liver (%)	4.35 ± 0.29	5.48 ± 0.48	5.64 ± 0.28	5.82 ± 0.36	0.06
Pancreas (%)	0.72 ± 0.03	0.74 ± 0.06	0.95 ± 0.07	0.82 ± 0.05	0.06
Proventriculus (%)	1.13 ± 0.05	1.20 ± 0.1	1.18 ± 0.1	1.17 ± 0.05	0.95
Gizzard (%)	3.80 ± 0.12	4.28 ± 0.44	4.79 ± 0.19	4.55 ± 0.53	0.30
Crop (%)	0.70 ± 0.03b	0.69 ± 0.05b	0.71 ± 0.07b	0.93 ± 0.03a	0.01
**42 days**					
Carcass (%)	62.19 ± 0.65	62.95 ± 0.75	62.04 ± 0.64	62.24 ± 1.40	0.33
Abdominal fat (%)	2.18 ± 0.21a	2.08 ± 0.18ab	2.04 ± 0.12ab	1.95 ± 0.19b	0.041
Liver (%)	3.09 ± 0.14	2.80 ± 0.12	2.99 ± 0.20	2.93 ± 0.08	0.56
Pancreas (%)	0.39± 0.03	0.44 ± 0.03	0.47 ± 0.03	0.39 ± 0.02	0.11
Proventriculus (%)	0.68 ± 0.04	0.68 ± 0.04	0.74 ± 0.06	0.69 ± 0.05	0.59
Gizzard (%)	2.73 ± 0.16	2.46 ± 0.09	2.34 ± 0.12	2.51 ± 0.25	0.33
Crop (%)	0.37 ± 0.02	0.31 ± 0.03	0.35 ± 0.01	0.41 ± 0.04	0.43

*Note*: Values in rows with different letters differ significantly (*p* ≤ 0.05).

^a^Results are mean ± SEM with *n* = 10 per treatment.

^b^All variables were analysed using Tukey's range test.

### 3.3 Immune System

The results of the effect of HPEE on the antibody titer response against SRBC, relative weight of lymphoid organs and immunoglobulin M and G titers are shown in Table 5. HPEE had no effect on the relative weight of the spleen and bursa of Fabricius and immunoglobulin G (IgG) titers. Broilers fed 450 mg/kg HPEE had higher total antibody titers against SRBC compared to the control group (*p* < 0.05); however, birds receiving 150 mg/kg HPEE had higher immunoglobulin M than the control (*p* < 0.05).

**TABLE 5:** Effect of experimental diets of ethanolic extract of *Hypericum perforatum* L. (HPEE) on serum antibody titer (log_2_) against SRBC, amount of IgM and IgG and weight of spleen and bursa of broilers at 24 and 42 days.^a^

Concentration of HPEE (ppm)					
Immune response	0	150	300	450	*p*‐value^b^
Antibody titer (log_2_) against SRBC	6.00 ± 0.28ab	6.50 ± 0.28ab	6.50 ± 0.28ab	7.50 ± 0.28a	0.04
IgM	4.50 ± 0.28b	5.75 ± 0.25a	5.25 ± 0.25ab	5.50 ± 0.28ab	0.03
IgG	2.00 ± 0.40	0.75 ± 0.25	1.25 ± 0.25	2.00 ± 0.40	0.06
**Bursa (weight in g)**					
24 days	0.44 ± 0.05	0.50 ± 0.08	0.46 ± 0.09	0.45 ± 0.07	0.95
42 days	0.24 ± 0.02	0.35 ± 0.01	0.33 ± 0.03	0.32 ± 0.04	0.06
**Spleen (weight in g)**					
24 days	0.20 ± 0.02	0.28 ± 0.05	0.25 ± 0.01	0.25 ± 0.03	0.45
42 days	0.26 ± 0.03	0.23 ± 0.01	0.21 ± 0.01	0.20 ± 0.03	0.62

*Note*: ^a,b^Values in rows with different letters differ significantly (*p* ≤ 0.05).

Abbreviations: IgG, immunoglobulin G; IgM, immunoglobulin M; SRBC, sheep red blood cell.

^a^Results are mean ± SEM with *n* = 10 per treatment.

^b^All variables were analysed using Tukey's test.

### 3.4 Serum Biochemical Indices

The effects of the experimental diets on blood indices of broiler chickens at 24 and 42 days of age are shown in Table 6. According to the results, serum cholesterol, LDL, HDL, glucose and triglycerides were not affected by HPEE at 24 days of age. HPEE affects serum total cholesterol, glucose and triglycerides at 42 days and reduces cholesterol, glucose and triglyceride concentrations (*p* < 0.05). The effect of dietary treatments on plasma liver enzyme activities such as aspartate aminotransferase (AST) and alanine aminotransferase (ALT) enzymes in broiler chickens at the ages of 24 and 42 days is presented in Table 7. The results showed that adding HPEE to the diet of broiler chickens could not significantly alter the activities of AST and ALT at 24 days; however, numerically, enzyme activity was reduced when birds were fed HPEE compared to the control. At 42 days, adding HPEE at 450 mg/kg to the diet caused a significant difference in AST enzyme activity compared to the control (*p* < 0.05). ALT enzyme activity also did not show significant differences among HPEE dietary treatments at 42 days. The diets containing 300 and 450 mg/kg HPEE reduced the concentration of plasma MDA as compared to control diet (Table 7). The lowest plasma MDA concentrations in blood and breast meat were observed in broilers fed 450 mg/kg HPEE compared to birds fed the control diet, which had the highest plasma and breast meat MDA concentrations.

**TABLE 6:** Effect of experimental diets of ethanolic extract of *Hypericum perforatum* L. (HPEE) on serum biochemical indices (mg/dL) of broilers at 24 and 42 days.^a^

Concentration of HPEE (ppm)					
Items	0	150	300	450	*p*‐value^b^
**24 days**					
Total cholesterol^c^	151.27 ± 1.73	150.60 ± 12.35	142.25 ± 12.53	146.23 ± 13.45	0.23
LDL‐cholesterol^c^	77.07 ± 1.55	72.56 ± 11.87	78.06 ± 3.63	75.36 ± 7.99	0.35
HDL‐cholesterol^c^	60.33 ± 2.82	63.30 ± 3.06	48.40 ± 9.19	56.30 ± 9.78	0.49
Triglyceride^c^	86.63 ± 13.13	92.15 ± 5.24	98.70 ± 2.15	91.05 ± 7.01	0.46
Glucose^c^	192.33 ± 6.94	194.33 ± 12.28	179.00 ± 30.31	210.00 ± 8.78	0.16
**42 days**					
Total cholesterol^c^	149.45 ± 3.40a	142.03 ± 7.30a	147.90 ± 7.86a	129.62 ± 3.53b	0.012
LDL‐cholesterol^c^	75.28 ± 2.14a	67.64 ± 4.83ab	74.41 ± 3.20a	57.17 ± 5.75b	0.041
HDL‐cholesterol^c^	60.27 ± 0.90	61.56 ± 4.28	60.67 ± 4.88	59.05 ± 7.02	0.382
Triglyceride^c^	86.82 ± 11.26	80.16 ± 2.14	80.05 ± 4.97	83.75 ± 4.86	0.471
Glucose^c^	182.50 ± 8.22a	149.67 ± 9.27b	152.75 ± 8.60b	162.00 ± 8.65b	0.038

*Note*: ^a,b^Values in rows with different letters differ significantly (*p* ≤ 0.05).

^a^Results are mean ± SEM with *n* = 10 per treatment.

^b^All variables were analysed using Tukey's range test.

^c^mg/100 mL.

**TABLE 7:** Effect of experimental diets of ethanolic extract of *Hypericum perforatum* L. (HPEE) on plasma liver enzyme activity and meat and plasma malondialdehyde (MDA) concentration.^a^

Concentration of HPEE (ppm)					
Items	0	150	300	450	*p*‐value^b^
Plasma MDA ng/mL	1.68 ± 0.05a	1.42 ± 0.04ab	1.31 ± 0.08b	1.26 ± 0.07b	0.003
Breast MDA ng/g	2.33 ± 0.017a	1.58 ± 0.021b	1.42 ± 0.016b	1.21 ± 0.014b	0.001
**Aspartate aminotransferase**					
24 days	161.50 ± 8.28	163.43 ± 7.92	138.13 ± 18.60	145.70 ± 17.45	0.53
42 days	218.60 ± 14.17a	172.80 ± 8.44ab	188.95 ± 9.89ab	163.35 ± 13.99b	0.03
**Alanine aminotransferase**					
24 days	12.66 ± 0.23	12.50 ± 0.28	12.25 ± 0.25	12.23 ± 0.47	0.46
42 days	12.50 ± 0.64	11.66 ± 0.23	12.00 ± 0.40	12.25 ± 0.62	0.29

*Note*: Values in rows with different letters differ significantly (*p* ≤ 0.05).

Abbreviation: MDA, malondialdehyde.

^a^Results are mean ± SEM with *n* = 10 per treatment.

^b^All variables were analysed using Tukey's range test.

## 4 Discussion

### 4.1 Chemical Composition

Analysis of HPEE showed that the main components in HPEE were α,β‐pinene, α‐meurolene, 1‐dodecanol, undecane, α‐sedrene, β‐ocimene, myrtenol and tridecane. Ghasemi‐Pirbalouti et al. (2014) reported that the main components present in Iranian (Charmahal‐Bakhtiari) *HPE* are α–pinene (12.5%), β‐pinene (8.3%), undecane (7%) and germacrene‐D (6.9%). In another research, the main components in Turkish *HPE* were reported to include α‐pinene (61.7%), 3‐careen (7.5%), β‐caryophyllene (5.5%), myrcene (3.6%), cadalene (3.2%) and β‐pinene (3%) (Cirak et al. 2010). These findings indicated that the components of *HPE* depend on its location. Therefore, the diversity in the bioactive components of *HPE* could have different effects of antioxidant, antibacterial and antiviral activity.

### 4.2 Performance and Carcass Traits

The results showed that feed intake, body weight gain and FCR were not affected by HPEE levels in broiler diets, which is consistent with previous studies (Feizi and Nazeri 2011; Seraji‐Kopkan et al. 2015). Landy et al. (2012) reported that supplementation with the antibiotic flavophospholipol improved the body weight of birds compared to birds fed *HPE*; however, they reported that the birds fed *HPE* had lower FCR and higher feed intake than birds fed flavophospholipol. *HPE* contained some anti‐nutritional factors such as tannins, which may have adverse effects on broiler production. Tannins bind to intestinal proteins and slow their absorption, which leads to reduced growth performance in poultry. In agreement with this finding, Toghyani et al. (2011) reported that broilers fed yarrow had no observable improvement in growth production due to the existence of anti‐nutritional agents such as tannins, hydrocyanic acid and linalool. Williams and Losa (2001) and Lovkova et al. (2001) showed that chemical compounds in medicinal herbs act as digestive stimulants by balancing the intestinal microbial ecosystem and stimulating the secretion of endogenous enzymes, increasing digestion and improving feed intake in broiler chickens. Furthermore, Landy et al. (2012) reported that high doses of *HPE* in poultry diets produced adverse effects on populations of beneficial gastrointestinal microflora, such as lactobacilli, and consequently reducing growth production. However, the main reasons for the lack of proper performance in last studies in broilers may be attributed to inappropriate use of the substance (high or low dosage) and inappropriate density and viscosity of the substance used (Nasiroleslami and Torki 2010; Grashorn 2010; Saleh et al. 2018).

Today in the poultry production industry, abdominal fat represents an undesirable index in broiler meat quality. This research showed that HPEE reduced the abdominal fat in the carcasses of broiler chickens. Similar to these results, Behroozilak et al. (2025) and Davoodi et al. (2014) observed that birds fed with *HPE* or HP powder had a lower abdominal fat percentage. Hence, the HPEE can lead to loss of abdominal fat. The relative reduction in carcass fat may be attributed to the bioactive compounds present in HPEE such as tannin. Tannin is one of the anti‐nutritional factors in HP that binds to proteins on the inner surface of the gastrointestinal tract which can inhibit absorption of nutrients and lead to reduced fat deposition in tissues. According to previous reports, HPEE had no differences in the relative weight of the intestine and digestive organs (Landy et al. 2012; Hernandez et al. 2004). Shoa Hassani et al. (2009) and Kianbakht (2010) reported that the lack of effect of HPEE on relative liver weight and the sharp increase in secretory activity and consequently, the increase in pancreas size could be the results of anti‐nutritional compounds present in *HPE* such as polyphenol compounds.

### 4.3 Immune System

The birds fed HPEE had a higher antibody titer against SRBC compared to the control group. In the poultry industry, one way to increase immunity is to reduce their susceptibility to infectious diseases (Liu 1999). In the present experiment, HPEE did not significantly affect lymphoid organ weight and IgG; however, HPEE diets significantly enhanced antibody titers against SRBC and immunoglobulin M (*p* < 0.05). Landy et al. (2012) reported that broilers fed HP had higher antibody titers against influenza virus. Oral inoculation of *Lactobacillus* to mice increased antibody titers against SRBC (Perdigon et al. 1995). The intestine and its microflora play a pivotal role in improving immune function (Noverr and Huffnagle 2004). Gram‐positive bacteria such as *Lactobacillus* and *Bifidobacterium* play an important role in improving the immune system function (Humphrey et al. 2002). The HPEE prevented Gram‐positive bacteria in the intestines of birds (Mazandarani et al. 2007). Other researchers have also reported that flavonoid‐rich plants can improve the immune function due to their antioxidant and vitamin C activity (Manach et al. 1996; Jafari et al., 2021). Hypericin and hyperforin in *HPE* may have anti‐inflammatory and antiviral effects in poultry, especially in broiler chickens (Behboodi et al. 2021; Ihan et al. 2024; Behroozilak et al. 2025). Polyphenols of *HPE* act as immunostimulants by mechanisms such as mononuclear phagocyte system, humoral and cellular immunity (Evstifeeva and Sibiriak 1996). In fact, there is a high correlation between the health conditions of farming environment, and the effect of extracts of plants (Vogt and Rauch 1991).

### 4.4 Serum Biochemical Indices

*HPE* had a significant effect on plasma biochemical traits at 42 days. In agreement with these results, Landy et al. (2012) reported that the addition of powdered *HPE* at a rate of 5 and 10 g per kg of broiler diets had no significant effect on total cholesterol, HDL, LDL and triglycerides concentration. The combined use of black cohosh and *HPE* had no significant effect on serum lipid profile except for HDL (Chung et al. 2007). The reducing effect of HP on cholesterol and atherosclerosis has also been confirmed in other studies (Behboodi et al. 2021; Ihan et al. 2024; Behroozilak et al. 2025). The anthocyanin compounds (water‐soluble flavonoids) in HP probably inhibit LDL oxidation by trapping oxygen in plasma and fluid between the vessel walls, exerting its anti‐atherogenic and antilipidemic effects (Yamakoshi et al. 1999). The reduction in blood glucose concentration due to the use of plant extracts can be attributed to the uptake of glucose by liver, fat and muscle, although this effect may be different from that of insulin (Richelle et al. 2004). In addition, AST and ALT activities are two indictors for evaluating liver function. Measuring of the activity of these enzymes is a useful test for the diagnosis of acute liver diseases such as hepatitis. ALT is a specific enzyme that increases only in liver disorders while AST increases during the liver parenchymal damage and cardiac or muscle damage. When the permeability of the liver cell membrane increases due to injuries, the release of these enzymes into the blood increases (Soochan et al. 2012; Hosseini‐Vashan and Piray 2021) and their activity increases. These findings are consistent with previous reports (Landy et al. 2012; Davoodi et al. 2014; Seraji‐Kopkan et al. 2015). However, HPEE supplementation in broiler diets resulted in reduced liver enzyme secretion by reducing liver damage. This could be a good indicator for assessing liver function.

### 4.5 Antioxidant Activity

The lowest MDA concentration in blood and breast meat were observed in the plasma of birds fed a diet containing 450 mg/kg HPEE. MDA induces the lipid peroxidation reaction, therefore, increasing MDA concentration has been shown to enhance lipid peroxidation. The diets containing higher levels of HPEE showed the highest antioxidant activity with the lowest lipid peroxidation in broilers. Bioactive compounds in HPEE, including hypericin, hyperforin, α, β‐pinene and phytol, have antioxidant activity and can strongly scavenge free radicals, leading to reduced cellular damage and MDA concentrations in plasma and meat (Kürekci et al. 2021; Behroozilak et al. 2025). However, some environmental stressors and increased dietary fat can increase free radical production and lipid peroxidation (Hosseini‐Vashan and Afzali 2008; Hosseini‐Vashan and Piray 2021). Altun et al. (2013) evaluated the antioxidant activity of aqueous, ethyl acetate and methanolic extract of *HPE* in vitro condition. Their results show that 2000 µg/mL of methanolic and ethyl acetate extracts is the highest level of antioxidant activity of *HPE*. In addition, the antioxidant activity of HP have been evaluated through DPPH radical scavenging assay, free radicals, superoxide anion radicals and ferric reducing antioxidant power (FRAP) tests. The antioxidant activity of *HPE* could be related to the high levels of flavonoid glycosides and bioflavonoids in the plant (Cirak et al. 2010). Seraji‐Kopkan et al. (2015) have also reported that adding aqueous extract of *HPE* to broiler diets reduced plasma MDA concentrations in broiler chickens.

## 5 Conclusions

It is concluded that HPEE had no significant effect on the growth performance and carcass characteristics in broiler chickens. Supplementation of HPEE to broiler diets can reduce cholesterol, LDL, glucose, abdominal fat and plasma enzyme activity of AST. Also, antioxidant status and immune response can be improved by reducing MDA concentration when HPEE is added to the diet.

## Author Contributions

**Hojjat Seraji‐Kopkan**: methodology, software, data curation, formal analysis, writing – original draft, data collection, statistical analyses, final revision, approval of article submission. **Seyyed Javad Hosseini‐Vashan**: conceptualization, methodology, data curation, supervision, project administration, writing – review and editing, writing – original draft, data collection, statistical analyses, final revision, approval of article submission. **Nazar Afzali**: supervision, project administration, writing – review and editing, data collection, statistical analyses, final revision, approval of article submission. **Mohammad Hassan Namaei**: methodology, formal analysis, validation, data collection, statistical analyses, final revision, approval of article submission.

## Funding

This research was supported by the University of Birjand (grant number: 32546).

## Ethics Statement

The present study followed the institutional guidelines for humane treatment of animals and was in accordance with the ethical regulations of the University of Birjand.

## Conflicts of Interest

The authors declare no conflicts of interest.

## Data Availability

The data that support the findings of this study are available on request from the corresponding author.
